# Generation of Face Privacy-Protected Images Based on the Diffusion Model

**DOI:** 10.3390/e26060479

**Published:** 2024-05-31

**Authors:** Xingyi You, Xiaohu Zhao, Yue Wang, Weiqing Sun

**Affiliations:** 1 National and Local Joint Engineering Laboratory of Internet Applied Technology on Mines, China University of Mining and Technology, Xuzhou 221008, China; 2 School of Information and Control Engineering, China University of Mining and Technology, Xuzhou 221008, China

**Keywords:** diffusion model, face privacy preserving, image generation

## Abstract

In light of growing concerns about the misuse of personal data resulting from the widespread use of artificial intelligence technology, it is necessary to implement robust privacy-protection methods. However, existing methods for protecting facial privacy suffer from issues such as poor visual quality, distortion and limited reusability. To tackle this challenge, we propose a novel approach called Diffusion Models for Face Privacy Protection (DIFP). Our method utilizes a face generator that is conditionally controlled and reality-guided to produce high-resolution encrypted faces that are photorealistic while preserving the naturalness and recoverability of the original facial information. We employ a two-stage training strategy to generate protected faces with guidance on identity and style, followed by an iterative technique for improving latent variables to enhance realism. Additionally, we introduce diffusion model denoising for identity recovery, which facilitates the removal of encryption and restoration of the original face when required. Experimental results demonstrate the effectiveness of our method in qualitative privacy protection, achieving high success rates in evading face-recognition tools and enabling near-perfect restoration of occluded faces.

## 1. Introduction

Rapid advances in information technology, including cloud computing, big data and artificial intelligence, have prompted researchers to pay more and more attention to privacy protection. [1]. Image processing and analysis technologies greatly facilitate tasks such as image recognition, but they also pose significant risks [2]. Almost all the time, some private photos and videos containing personal facial information are transmitted to the cloud or used by various organizations [3]. Privacy refers to very important personal facial feature information. Nevertheless, facial information is considered sensitive due to its inclusion of personal details. If not adequately protected [4,5,6], private data with facial features can be illegally obtained, possessed and exploited to cause unnecessary losses. Therefore, there is a pressing need for technology that ensures the safety of facial privacy data while performing routine image analysis and recognition tasks.

Early work on facial privacy preservation primarily involved techniques such as deliberate masking [7], blurring [8] and pixelation [9]. However, these methods directly remove facial information, resulting in compromised visual quality and reduced reusability. Subsequently, researchers shifted towards approaches centered on facial replacement [10,11,12,13,14,15,16]. This strategy aims to obfuscate the identity of the original face by substituting parts of it while retaining some facial features to maintain usability. Nevertheless, these methods often produce images with noticeable artifacts that lack a realistic appearance. Moreover, existing research tends to focus on specific protection types, leading to evident edit traces, poor realism and limited recovery capabilities. Through analyzing the above methods, it can be found that while existing facial privacy-protection methods effectively prevent malicious third parties and illegal users from accessing original facial information, they suffer from limitations. These methods often result in a loss of the original facial details and fail to consider the authenticity of the replaced face. Consequently, the occluded face appears unnatural and easily detectable, posing a heightened risk to privacy. As emerging application scenarios demand more comprehensive solutions, there is a need for advanced facial privacy-protection techniques that address these shortcomings. Our method is able to prevent facial-recognition tools from recognizing the protected facial information, generate natural recoverable face privacy-preserving images and generate diverse encrypted images, increasing the strength of privacy protection.

As societal norms evolve, facial information is increasingly exposed on public platforms and social media posts may be used for undesirable political targeting [17], necessitating comprehensive approaches to protect facial privacy. Beyond mere protection, there is a growing need for techniques that enable high-fidelity reduction of original facial images to meet diverse user requirements. For instance, on social media platforms, users desire natural-looking photos that safeguard their facial privacy from unauthorized access while allowing authorized individuals, such as friends or family, to view the original face. Machine learning models may reveal sensitive information about the data used for training, and public databases may be deanonymized with only a few queries [18]. Furthermore, in surveillance contexts, preserving the original facial details of captured individuals is essential for criminal identification and post-privacy protection. Therefore, urgent efforts are required to develop methodologies that effectively address various facial privacy-protection requirements and mitigate the limitations of existing approaches while also improving the recoverability of facial information.

Innovative solutions are necessary to address two key challenges in emerging application scenarios for facial privacy protection. Firstly, natural-looking facial privacy protection must be achieved to avoid raising suspicion while effectively concealing sensitive facial features. Secondly, reversibility must be ensured, allowing occluded images to be seamlessly restored to their original state. Leveraging the powerful generation capabilities of diffusion models, we propose a novel approach for facial privacy protection called the diffusion-based facial privacy-protection network (DIFP). DIFP is designed to provide flexible and secure facial privacy protection while enabling identity recovery when necessary. During the process of concealing visual identity cues, DIFP generates highly realistic facial information that is distinct from the original face. Additionally, our method ensures excellent recovery quality and offers several advantages, including enhanced security by effectively concealing personally identifiable information; practicality by preserving usability for downstream computer vision tasks like face detection; diversity by generating a range of encrypted images from a single original image; and environmental invariance by maintaining identity-independent attributes such as background and pose. Extensive experiments conducted on publicly available facial datasets validate the effectiveness of our proposed method in achieving natural-looking facial privacy protection with seamless reversibility.

The main contributions of this paper are as follows:

(1) We propose a novel diffusion-based method for facial privacy protection, offering precise control over the identity and style of generated privacy images while ensuring lossless restoration of the original face when required.

(2) By utilizing a classifier-free guidance mechanism, we encode the conditions of identity and appearance style for the input face. We employ a two-stage training strategy to generate privacy images with customized identity and style guidance, thereby enhancing the customization and versatility of the generation model.

(3) We employ an iterative technique to improve the realism of generated images by adjusting the realism scale parameter. Enhanced diversity in privacy images is achieved by leveraging differences in generated facial identities, enabling flexible and controllable adjustments to appearance and realistic details.

(4) Experimental results demonstrate the superiority of our method over existing approaches, as it significantly alters facial features while maintaining photorealism, resulting in high-quality restoration outcomes.

## 2. Related Work

### 2.1. Diffusion Model

Diffusion-based generative models (DMs) have emerged as powerful tools for modeling and generating complex data, with notable advancements in density estimation and sample quality [19,20,21,22,23]. Early approaches relied on Markov chains, necessitating multiple iterations to generate high-quality samples [14]. Subsequent innovations, such as the Deterministic Diffusion-based Image Modeling (DDIM) technique [21], have significantly reduced sample generation time. While the incorporation of category information into diffusion models has shown promise for improving sample realism [8], this approach often incurs high training costs due to the need for additional classifiers. To address this limitation, the classifier-free diffusion method [15] combines conditional and unconditional diffusion models to balance sample quality and diversity. Furthermore, efforts to enhance training and inference efficiency include denoising in the latent space of pre-trained autoencoders, reducing computational requirements. Presently, diffusion models find applications across various computer vision tasks.

### 2.2. Privacy-Protection Model

Hsu et al. [24] identified and processed sensitive features in the data by estimating the information density of each feature. In the process of generating or transforming data, privacy leakage is controlled while ensuring that the data still have a certain degree of utility. The use of information bottleneck and information funnel theory to facilitate invariant representation learning is a key aspect in the field of machine learning and deep learning, especially in computer vision and pattern-recognition tasks. Freitas and Geiger et al. [25] use information bottleneck and information funnel theory to promote invariant representation learning so as to balance the contradiction between privacy protection and data utility. Huang and Hesham et al. [26] proposed an efficient algorithm to solve the private funnel optimization problem with DC structure by using the characteristics of differential convex functions, which may have a significant improvement in convergence speed or solution quality compared with traditional methods. Razeghi et al. [27] used information theoretic principles, in particular the privacy funnel model (PF), which is a method to quantify the trade-off between information obfuscation and data utility in data protection. The privacy funnel model protects data by minimizing the leakage of sensitive information through self-information loss, that is, logarithmic loss, while maximizing the retention of information for legitimate purposes.

### 2.3. Privacy Protection in Face Recognition

Face privacy protection refers to the protection of important facial information through some specific methods to prevent illegal acquisition, recognition, and use. Morales et al. [28] proposed a privacy-preserving feature representation learning approach that aims to eliminate sensitive information, such as gender or ethnicity, from the learned representation while maintaining data utility. This approach centers on adversarial regularization to remove sensitive information from the learning objective. Tran et al. [29] introduced disentangled representation learning generative adversarial networks (DR-GAN) to address the challenge of pose variation in face recognition. Unlike traditional approaches that generate frontal faces from non-frontal images or learn pose-invariant features, DR-GAN performs both tasks jointly via an encoder-decoder generator structure. This enables it to synthesize identity-preserving faces with arbitrary poses while learning discriminative representations. Gong et al. [30] proposed an approach to mitigate bias in automatic face recognition and demographic attribute estimation algorithms, with a focus on addressing observed differences in performance between different demographic groups. We leverage adversarial learning to extract decoupled feature representations of identity and demographic attributes (gender, age and race) and minimize bias by reducing the correlation between these feature factors to enhance the robustness and accuracy of face representations for different demographic groups. Park et al. [31] propose a fair-aware disentangled variational autoencoder (FD-VAE) that aims to mitigate discriminatory outcomes related to protected attributes such as gender and age in AI systems without sacrificing beneficial information for the target task. We propose a decorrelation loss to properly align the information in these subspaces, focusing on preserving information useful for the target task while excluding protected attribute information. Li et al. [32] introduced debiasing alternating networks (DebiAN) to mitigate bias in depth image classifiers without the need for protected attribute labels, aiming to overcome the limitations of previous approaches that required full supervision. We demonstrate the effectiveness of DebiAN through experiments on both synthetic datasets, such as multi-color MNIST, and real-world datasets, showing its ability to discover and improve bias mitigation. Suwała et al. [33] introduce PluGeN4Faces, a plugin for StyleGAN that aims to manipulate facial attributes such as expression, hairstyle, pose and age in images while preserving the person’s identity. It uses contrastive loss to closely cluster images of the same individual in the latent space, ensuring that the change of attributes does not affect other features such as identity.

However, these methods often rely on high-quality face datasets for training and cannot achieve satisfactory results in terms of privacy and quality of recovered images. Therefore, our method adopts a new diffusion model-based facial privacy-protection method, which can effectively achieve facial privacy protection and face recovery while ensuring image clarity.

## 3. Method

**Figure 1:**
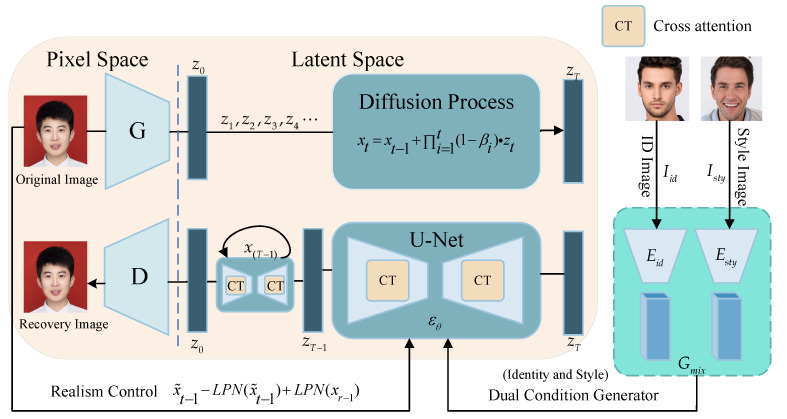
The overall framework of the face privacy-protection model (DIFP).

A Face Privacy-Protection Model (DIFP) based on a diffusion model is proposed. As shown in Figure 1, in the training process, a high-quality ID image is obtained from the training dataset, from which an identity code $I_{id}$ is derived. Additionally, an image of any style is selected to obtain the style code $I_{sty}$. These codes are then combined through a dual-conditional face data generator guided by identity and style. Realism control is added to the original image through iterative latent variable refinement technology to achieve more realistic privacy protection. Finally, the initial latent encoding $z_{0}$ is obtained by denoising the noisy latent encoding $z_{T}$, and it is decoded to perform identity recovery. Section 3.1 introduces the problem formulation and the threat model. The fundamentals of the diffusion model are briefly discussed in Section 3.2. Finally, this is followed by an in-depth exploration of face privacy protection in Section 3.3.

### 3.1. Preliminaries

#### 3.1.1. Problem Formulation

Let us have an original dataset of facial images $D={\left\{x_{i}\right\}}_{i=1}^{N}$, where $x_{i}$ denotes the facial image of the *i*th individual, and *N* represents the total number of individuals in the dataset. The objective is to devise a privacy preservation mechanism DIFP, underpinned by diffusion models, that transforms original facial images $x_{0}$ into privacy-preserving synthetic images $x^{\prime }$, all the while maintaining a degree of utility for recognition purposes.

Diffusion models are conventionally defined as a sequence of noise-injection steps progressing from a clean data distribution $p(x_{0})$ to a noise distribution $p(z_{t})$, with $z_{t}$ approximating Gaussian noise. Conversely, in privacy-protection applications, we use the inverse process to approximate from noise to clear data distribution, but in this process, we introduce privacy-protection mechanisms according to the characteristics of the threat model. For the best privacy protection, we use anonymization privacy protection, so that the generated $x^{\prime }$ looks similar to the original input but no longer corresponds to any particular original individual at all. At the same time, we also consider data practicality and retain key information for face recovery when necessary.

Privacy preservation aims to minimize information leakage $I(x_{0};x^{\prime })$, the mutual information between the original image $x_{0}$ and the generated image $x^{\prime }$. Meanwhile, to ensure a certain level of recognition performance, the utility of the generated images for legitimate recognition tasks, quantifiable as the accuracy of the conditional probability $P(x|x^{\prime })$ where *x* corresponds to the associated label (such as identity), needs to be maximized.

Thus, the core optimization goal encapsulates a trade-off problem:

(1) $$min_{P(x^{\prime }|x_{0})}\left(\lambda I\left(x_{0};x^{\prime }\right)-U\left(x,x^{\prime }\right)\right)$$

Here, λ acts as a balancing parameter, modulating the compromise between privacy protection and utility; $U(x,x^{\prime })$ signifies a function gauging the utility of recognition, being a recognition accuracy metric, and $I(x_{0};x^{\prime })$ denotes the magnitude of privacy leakage, computable through mutual information theory.

#### 3.1.2. Threat Model

The threat model refers to the challenges faced by the privacy-protection-enhancement technology we designed, including various types of attacks that try to break its integrity and confidentiality. We construct the threat model from several aspects, such as the adversary objectives, the adversary knowledge level, the objectives of privacy protection and the practicality of the model.

Adversary objectives: the opponents of sensitive facial features in the initial image $x_{0}$, usually associated with personal identity privacy.

Data-access and diffusion model relationships: rivals access to public $x^{\prime }$; this means that the opponent can see the data of some form of output, or it is designed to protect privacy.

Adversary knowledge level: opponents may know the principle of diffusion model $p(z_{t-1}|z_{t})$ before the moment of data associated with the noise of the moment after joining.

Privacy-protection objectives: under the threat model, the privacy-protection objectives are to design a model, make it practical with respect to meeting the data $x_{0}$ (utility), and at the same time minimize information about the characteristics of leakage, and to find a balance between protecting personal privacy and maintaining image utility.

The practicability of the model: the threat model is designed to generate types. The privacy-protection method provides a framework, allowing one to, in the case of known rival abilities and behaviors, develop an effective privacy policy. Through the definition of this threat model, we can consider the potential rival attack means and strategy and make sure they match the proposed privacy-protection method.

### 3.2. Overview of Generative Diffusion Models

The diffusion model, a probabilistic generative model, learns the data distribution p(x) by reverse-learning a Markov chain with a fixed length *T* and gradually denoising a normally distributed variable. It generates target data by incrementally introducing random noise into the input data. This process consists of two stages: forward diffusion and backward reconstruction. First, a pre-trained encoder *G* extracts a latent encoding $z_{0}$ from the initial image $x_{0}$. Subsequently, denoising operations are performed in the latent space, followed by decoding the feature vector into the desired image using the pre-trained decoder *D*. This process encompasses both encryption and recovery of images for our face privacy-protection model.

During the forward diffusion phase, random noise is incrementally added to the initial image in order to progressively align the data with the target data. This process, also known as a fixed-length Markov chain, involves generating a sample $z_{t}$ by introducing noise ϵ at each time step *t* given the initial data $z_{0}$. The diffusion process can be mathematically represented by Equation (2).

(2) $$z_{t}=z_{t-1}+\prod_{i=1}^{t}\left(1-\beta_{i}\right)\cdot \epsilon_{t},$$

where $z_{t}$ denotes the current data at time t, $z_{t-1}$ denotes the previous data at time t−1, the data distribution at time t is related to the data at time t−1, and the addition of noise, $\prod_{i=1}^{t}\left(1-\beta_{i}\right)\cdot \epsilon_{t}$ denotes the added noise, $\epsilon_{t}$ is the introduced random noise, $\beta_{i}\in \left(0,1\right),\beta_{1}<\beta_{2}\cdots <\beta_{T}$ is the diffusion coefficient at time step t, and determines the size of the introduced noise, which is usually uncertain, according to the needs of the task. As time steps increase, we introduce smaller noise in the early stages of diffusion and larger noise in the later stages. The value of $\beta_{i}\in \left(0,1\right),\beta_{1}<\beta_{2}\cdots <\beta_{T}$ can be either a learnable parameter of the model or a manually set hyperparameter, that the image data at time t generated under the condition at time t−1 is subject to the standard normal distribution, as shown in Equation (3).

(3) $$q\left(z_{t}|z_{t-1}\right)=N\left(z_{t};\left(1-\beta_{t}\right)z_{t-1},\beta_{t}I\right),$$

where $q(z_{t}|z_{t-1})$ denote the conditional probability distribution of the data $z_{t}$ at the current time step *t*, given the data $z_{t-1}$ at the previous time step t−1. Normal distribution N is a continuous probability distribution. $z_{t}$ denotes the data at time step *t*, $1-\beta_{t}$ is a coefficient that regulates the increase in noise in the diffusion process at each step, thereby adjusting $z_{t-1}$ its impact $z_{t}$. $z_{t-1}$ denote the data at time step t−1, representing the noisy data from the previous time step. $\beta_{t}I$ denotes the Gaussian noise that is added to each step of the diffusion process, and its variance is controlled by $\beta_{t}$ the identity matrix *I*. t∼[1,T] denotes the time step *t* from 1 to *T*, where *T* is the total number of steps of the diffusion process.

In the backward reconstruction stage, which is initiated from the final outcome of diffusion, gradual denoising is employed to restore the data to its target form. A decoder *D* predicts the mean and covariance of $z_{t-1}$ for a given input $z_{t}$. The model is trained using the standard Mean Square Error (MSE) loss to predict the added noise, represented by $\epsilon_{\theta }\left(z_{t},t\right)$ using the parameterized Gaussian transfer Equation (4).

(4) $$p\left(z_{t-1}|z_{t}\right)=N\left(z_{t-1};\mu_{\theta }\left(z_{t},t\right)\sum_{\theta }\left(z_{t},t\right)\right),$$

where $p(z_{t-1}|z_{t})$ denotes the conditional probability distribution of the data $z_{t-1}$ at the previous time step t−1, given the data $z_{t}$ at the current time step *t*, in the reverse denoising process. The θ denotes the model parameters. N represents the normal distribution, a continuous probability distribution. $z_{t-1}$ denote the data at time step t−1, $z_{t}$ denote the data at time step *t* and $\mu_{\theta }\left(z_{t},t\right)$ denote the mean of the conditional distribution, which is a function of $z_{t}$ and time step *t*, computed from a network model with the network parameterized as θ. $\sum_{\theta }\left(z_{t},t\right)$ denotes the covariance matrix of the conditional distribution, a function of $z_{t}$ and time step *t*, also computed from the network model.

The mean $\mu_{\theta }\left(z_{t},t\right)$ can be obtained either through Bayes’ theorem or by predicting the noise $\epsilon_{\theta }\left(z_{t},t\right)$ using Equation (5). The weight $\alpha_{t}$ in Equation (6) represents the impact of gradually adding noise. The covariance $\sum_{\theta }\left(z_{t},t\right)$ incorporates an additional loss term $L_{v}$ (Equation (7)) to maximize the likelihood of the model while minimizing the KL divergence between the distribution of the eigenvariate and the prior distribution. This process interpolates between the upper and lower bounds of the original fixed covariance, facilitating a smoother approximation of the true distribution during learning.

(5) $$\mu_{\theta }\left(z_{t},t\right)=\frac{1}{\sqrt{\alpha_{t}}}\left(z_{t}-\beta_{t}\sqrt{1-\bar{\alpha_{t}}}\epsilon_{\theta }\left(z_{t},t\right)\right),$$

where $\mu_{\theta }\left(z_{t},t\right)$ represent the predicted data mean of the previous time step in the backward process, considering the current noisy data $z_{t}$ at time step *t*. μ denote the mean and θ denote the model parameters. $z_{t}$ denotes the data at time step *t*, and $\alpha_{t}$ is a predetermined scheduling parameter, typically between 0 and 1, that controls the amount of noise added from $z_{t-1}$ to $z_{t}$. $\alpha_{t}$ is the coefficient associated with $\beta_{t}$ controlling the addition of noise. $\bar{\alpha_{t}}$ denote the $\alpha_{i}$ cumulative product from time step 1 to *t*. The model parameters determine $\epsilon_{\theta }\left(z_{t},t\right)$, a random variable that represents the predicted value of the noise term at time step *t*. ϵ signify the addition of noise to the data at time step *t*.

(6) $$\bar{\alpha_{t}}=\prod_{i=1}^{t}\left(1-\beta_{i}\right),\beta_{i}\in \left(0,1\right),$$

Equation (6) shows the $\bar{\alpha_{t}}$ calculation method in Equation (5), which ensures the smoothness of the forward diffusion process and the accuracy of the backward denoising process, as well as the flexibility of designing the diffusion process. By considering $\bar{\alpha_{t}}$ and $\beta_{t}$, the noise growth in each step can be more finely controlled. $\bar{\alpha_{t}}$ captures the cumulative effect of noise from the start to the current step, while $\beta_{t}$ focuses on noise addition at the current step, providing precise prediction and noise removal for the reverse denoising process.

(7) $$L_{v}=R_{q(z)}\left[log\;p\left(x|z\right)\right]-KL\left(q\left(z\right)∥p\left(z\right)\right),$$

where “∥” represents the calculation of KL divergence, which is used to measure the similarity between the probability distribution of the real data and that of the model-generated data. “N” stands for expectation operation, which describes the expectation with respect to the distribution q(z). q(z) represents the distribution of the eigenvariables, p(x|z) denotes the generated data distribution, and KL refers to the divergence measure known as KL divergence. The removal of back-guidance noise facilitates the gradual restoration of the data to its initial state.

Our face privacy-protection model achieves the gradual generation of complex privacy-protected images that resemble the target image from a simple input face image. This is accomplished through noise guidance and control, along with the gradual reduction of noise, ultimately aiming to closely approximate the original face image.

### 3.3. Framework for Face Privacy Protection

According to the adversary’s ability defined in the threat model, we use differential privacy to formulate a guidance strategy for adding identity and style conditions to the diffusion model and obtain a rich and diverse anonymized face image. Although the adversary knows the working principle of the basic diffusion model, only the interference image can be obtained after denoising. We assume that the adversary model is not available, which makes our method both confidential and practical to recover the face image and perform the corresponding task when necessary.

#### 3.3.1. Diffusion Models Guided by Identity and Style Conditions

**Figure 2:**
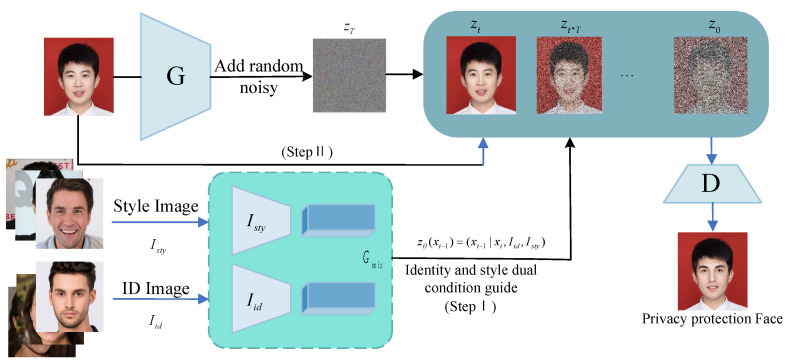
Identity and style conditions guide the generation process of face privacy-preserving images.

In our approach, we utilize the U-Net structure, which is a fundamental component of the diffusion model, for post-hoc prediction. It takes inputs such as the current time step $z_{t}$ and the associated conditional identity $I_{id}$ and style $I_{sty}$. Modifications are made to the parameter changes of the mean $\mu_{\theta }$ and covariance $\sum_{\theta }$ during the state transition from one time step to the next, incorporating conditions to facilitate conditional generation control. As shown in Figure 2, this enhances both flexibility and controllability of the generated results. By adjusting the level of conditional guidance, it tailors the resulting images more towards specific identity and style attributes rather than solely relying on random noise. The conditionally generated Gaussian transitions are represented by the following Equation (8). To model the reverse process in a diffusion model, we use identity and style as conditional inputs to make the model take into account the details of identity and style when generating images, thus generating what we hope will be more accurate and consistent images.

(8) $$p_{\theta }^{\prime }\left(z_{t-1}|z_{t},I_{id},I_{sty}\right)=N\left(z_{t-1};\mu_{\theta }\left(z_{t},t,I_{id},I_{sty}\right),\sum_{\theta }\left(z_{t},t,I_{id},I_{sty}\right)\right),$$

where $p_{\theta }^{\prime }\left(z_{t-1}|z_{t},I_{id},I_{sty}\right)$ represents the probability density function that generates the previous time step state $z_{t-1}$ based on the conditions $I_{id}$ and $I_{sty}$. The parameters $\mu_{\theta }$ and $\sum_{\theta }$ correspond to the adjusted mean and covariance parameters, respectively, which are influenced by the identity and style conditions.

In practice, similar to previous research, the conditional denoising process is trained to predict noise while incorporating additional identity and style information, represented as $\epsilon_{\theta }^{\prime }\left(z_{t},t,I_{id},I_{sty}\right)$.

To regulate the influence of identity and style conditions, we adopt a classifier-free guidance method that allows the generative model to be guided without relying on specific classifier labels. This enables conditioning the model during generation without requiring category information. The adjustment of dual-conditional guidance is achieved by introducing conditional vectors into the generation process. We employ a two-stage training strategy, segregating the training process into distinct phases with different objectives and configurations, as expressed by Equation (8). The initial stage trains the model with complete identity and style conditions to expedite its learning of condition correlations from the beginning, ensuring a robust starting point. During this phase, the loss function encompasses generating complete conditions, as expressed by Equation (9), used to optimize model parameters during training to better predict noisy data, it measures the difference between the noise $\epsilon_{\theta }^{\prime }$ predicted by the model and the actual noise ϵ. By minimizing a loss function, the model learns how to recover data from noisy data while taking into account the guiding information of identity and style, which can help generate images that are consistent with the identity and style of the input.

(9) $$L_{sim}^{\prime }=R_{t,z_{0},\epsilon }[∥\epsilon -\epsilon_{\theta }^{\prime }\left(z_{t},t,I_{id},I_{sty}\right){∥}_{2}],$$

In the loss function, ϵ represents the actual noise term, while the noise term predicted by the model $\epsilon_{\theta }^{\prime }\left(z_{t},t,I_{id},I_{sty}\right)$ is influenced by identity and style conditions. The L2 norm ${∥\cdot ∥}_{2}$ signifies the Euclidean distance between two vectors, representing expectations for all time steps *t*, initial image $z_{0}$ and noise term ϵ in $R_{t,z_{0},\epsilon }$. This formulation enables the model to generate outputs closely resembling actual noise terms during the initial stage of training, facilitating correct learning of correlations with identity and style conditions.

The model’s performance is enhanced in the second stage through iterative refinement and optimization of its parameters. During this stage, the model undergoes further training with a subset of conditions, where only 40% of the original pixels associated with each condition are randomly replaced to augment the training data. This process helps achieve unconditional representation and enhances the model’s ability to generate outputs across a wider range of conditions, thereby improving its generalization capabilities. By employing two distinct training phases, we can effectively guide the model’s learning process, allowing it to focus on specific objectives and tasks at different stages. In the sampling phase, Equation (10) is used to regulate the balance between identity and style fidelity, facilitating flexible guidance of the generation process without requiring specific classifier labels. This methodology enhances the adaptability and utility of the generative model.

During generation, the model computes a base unconditional noise term $\epsilon_{\theta }^{\prime }\left(z_{t},t,⊘,⊘\right)$ and then adds the influence of the identity and style conditions back into the generation process, respectively, depending on the values of $s_{id}$ and $s_{sty}$. By adjusting the values of $s_{id}$ and $s_{sty}$, we can control the influence of the input identity and style conditions on the generated image.

(10) $$\epsilon_{\theta }^{\prime }\left(z_{t},t,I_{id},I_{sty}\right)=\epsilon_{\theta }^{\prime }\left(z_{t},t,⊘,⊘\right)+s_{id}\left(\epsilon_{\theta }^{\prime }\left(z_{t},t,I_{id},⊘\right)-\epsilon_{\theta }^{\prime }\left(z_{t},t,⊘,⊘\right)\right)+s_{sty}\left(\epsilon_{\theta }^{\prime }\left(z_{t},t,⊘,I_{sty}\right)-\epsilon_{\theta }^{\prime }\left(z_{t},t,⊘,⊘\right)\right)$$

where $\epsilon_{\theta }^{\prime }\left(z_{t},t,I_{id},I_{sty}\right)$ is a final noise term that depends on the current time step t and the current noisy data $z_{t}$, as well as the identity condition $I_{id}$ and style condition $I_{id}$; θ denotes the model parameters; ⊘ is used to generate unconditional noise terms that do not depend on identity or style. $s_{id}$ and $s_{id}$ are control parameters to balance the influence of identity and style on the generation process. $\epsilon_{\theta }^{\prime }\left(z_{t},t,I_{id},⊘\right)$ and $\epsilon_{\theta }^{\prime }(z_{t},t,⊘,I_{sty}$ denote the noise terms in the identity-only and style-only conditions, respectively. $\epsilon_{\theta }^{\prime }\left(z_{t},t,⊘,⊘\right)$ denotes the noise term without the influence of identity or style conditions.

During the second stage, the model utilizes partial conditions and modifies the loss function to incorporate the portion generated unconditionally. Equation (11) displays the loss function.

(11) $$L_{par}^{\prime }=R_{t,z_{0},\epsilon }[∥\epsilon -\epsilon_{\theta }^{\prime }\left(z_{t},t,⊘,⊘\right){∥}_{2}],$$

where R represents the expected operation; *t* is the time step of the diffusion process; $z_{0}$ represents the input data without noise; ϵ denotes the actual noise term added to the data $z_{0}$ at time step *t*, while the noise term predicted by the model $\epsilon_{\theta }^{\prime }\left(z_{t},t,⊘,⊘\right)$ is influenced but without considering identity and style conditions. ${∥\cdot ∥}_{2}$ signifies the L2 norm, indicating the Euclidean distance between the two vectors. $R_{t,z_{0},\epsilon }$ represents expectations for all time steps, initial image and noise term. During the second phase of training, the objective is to refine the model’s generation outcomes to closely align with the actual noise term while disregarding identity and style conditions. This enhances the model’s capacity for generalization across a broader range of scenarios and improves its ability to generate diverse privacy-protected images in a more controlled manner.

Our training strategy incorporates a condition-controlled approach to guide the diffusion model, allowing for different levels of guidance, including full, partial and no guidance conditions. This enhances the model’s ability to generate a diverse range of facial images while ensuring privacy protection. By adjusting the level of conditional guidance, we can personalize the generated images in terms of identity and style, thereby increasing their diversity and expressive potential.

#### 3.3.2. Realistic Control

To address concerns regarding the authenticity and trustworthiness of privately generated facial images, we propose a mechanism that ensures reliability and realism in the output. Our objective is to modify only the visual attributes of the face while maintaining consistency with other contextual factors, thereby guaranteeing the authenticity of private images. We present a three-fold control approach that integrates identity and style guidance alongside realism control. This technique aims to generate private images that closely resemble their original counterparts while adhering to conditional constraints. Specifically, we introduce realism control by iteratively refining latent features and employing two-dimensional classifier-free guidance for low-pass filtering downsampling on the initial image. These additional adjustments enhance the generation process, resulting in greater realism and authenticity in the produced images.

During the inference stage of the generative model, it is crucial to enhance the quality of generated privacy images and ensure they meet specific conditions by refining latent features. To achieve this, we propose an iterative method for refining latent features in the generated image. This method involves multiple rounds of adjustments to iteratively improve the image quality while maintaining consistency with the provided identity and style information. We accomplish this by progressively refining the latent variables using a downsampled original image at each intermediate transformation stage of the generation process. The objective is to align the generated privacy-preserving face images more accurately with user-provided input or adhere closely to the data distribution of the original image.

To enhance the authenticity of the generated images while preserving identity and style details, we employ a linear low-pass filtering technique (LP) to reduce image resolution and minimize high-frequency elements. Subsequently, the filtered image is downscaled to a size denoted by *H* and then upscaled back to its original resolution. The value of *H* is determined based on the realism scale parameter, and a reference image containing both identity and style information is used for fine-tuning the resulting image’s consistency with the reference. As shown in Equation (12), the realism of the image is controlled by the iterative latent variable refinement method in the generation process, which combines the predicted data $x_{t-1}^{\prime }$ and the reference image $X_{com-1}$ processed by the low-pass filter to generate the final image $x_{t-1}$.

(12) $$x_{t-1}=x_{t-1}^{\prime }-LPH\left(x_{t-1}^{\prime }\right)+LPH\left(X_{com-1}\right),$$

where $x_{t-1}$ represents the final generated data at time step t−1, which is computed by combining $x_{t-1}^{\prime }$ with a reference image $X_{com-1}$. LPH(·) denotes a linear low-pass filter operation that downsamples the input image to a transform size *H* and then upsamples it back to the original size. This process helps to adjust the high and low frequency content of the image, thus controlling the detail and realism of the image. $X_{com-1}$ denotes the reference image at time step t−1, which is obtained by gradually injecting noise into the input identity and style combined image $X_{com}$. This reference image contains identity and style information that is used to guide the generation process.

The realism scale factor adjusts the similarity between the resulting image and the original image, ensuring that the generated image maintains authenticity while closely matching the appearance and style of real image data distribution. This is achieved by controlling specific image parameters, as indicated in Equation (13). $g_{rea}$ and *M* determine the value *H* to control the scale of the low-pass filter LP’s downsampling and upsampling operations, which are part of the iterative latent variable refinement process to adjust the image’s detail and realism during generation. By manipulating the realism scale factor, the generation quality of privacy-protected face images can be fine-tuned, dynamically balancing condition control and image realism during the generation process to yield more realistic privacy images.

(13) $$H=-g_{rea}\left(M-1\right)+\left(M+k\right),$$

where *H* represents the parameter determining the size of the image transformation (downsampled size), while $g_{rea}$ denotes the realism scale, controlling the balance between consistency and realism during image generation. As shown in Equation (14), as $g_{rea}$ approaches 0, *H* increases, preserving more high-frequency information and detail akin to the original image, albeit with potential distortion. Conversely, as $g_{rea}$ approaches 1, *H* decreases, resulting in a smoother image more aligned with the target data distribution. Here, *M* denotes the size of the reference image $X_{com}$, and *k* represents a constant term.

(14) $$g_{rea}=\frac{SSIM(x_{t-1}^{\prime },X_{com-1})}{SSIM(x_{t-1}^{\prime },x_{t-1}^{\prime })},$$

where $x_{t-1}^{\prime }$ is the generated image, $X_{com-1}$ is the reference image and SSIM is the structural similarity index. $g_{rea}$ represents the similarity between the generated image and the reference image. If the similarity between the generated image and the reference image is higher, the value of $g_{rea}$ will be closer to 0. If the similarity between them is low, the value of $g_{rea}$ is close to 1.

#### 3.3.3. Face Identity Recovery

In this section, encoder *G* is used to extract key features from the original image and convert it into latent space $z_{0}$, preserving essential original information $F_{e}$ that represents the image’s latent space representation for face image restoration purposes. During the privacy-protection process described in Section 3.2, noise is added to the initial latent code $z_{0}$ to obtain the latent code $z_{t}$. Subsequently, using inversion [34], a map containing noise serves as the key information $F_{i}$. Equation (15) calculates the time steps for noise addition, determining when noise should be introduced throughout the privacy-protection process. Adding noise at this stage helps preserve the global structure and characteristics of the image while obscuring facial appearance, making facial features less visually discernible and effectively concealing individual identity.

(15) $$t=S_{n}*T,$$

where *T* represents the total number of steps involved in noise removal, while $S_{n}$ denotes the scaling factor responsible for regulating the intensity of noise. This scaling factor allows for adjusting the noise intensity, thereby influencing the extent of noise introduction and removal. The choice of time step directly impacts the intensity of noise manipulation.

During the face restoration process, an iterative update of the noise image $z_{t}$ produces the denoised face restoration image. Equation (16), the key step in generating the face restoration image, utilizes the conditional embedding of the original image’s key information to restore the noisy image. Specifically, an updated latent representation $z_{t-1}$ corresponding to the denoised recovered image at time step t−1 is generated by combining the noisy image $z_{t}$ and the noise term $\epsilon_{\theta }^{\prime }\left(z_{t},t,F_{e},F_{i}\right)$ with the key information of the original image. We repeat this process until we generate the final noise-free image. This way, the diffusion model gradually recovers high-quality face images from noise while preserving the details of the original data distribution.

(16) $$z_{t-1}=\sqrt{\frac{\alpha_{t-1}}{\alpha_{t}}}z_{t}+\left(1-\sqrt{1-\alpha_{t-1}}\right)\epsilon_{\theta }^{\prime }\left(z_{t},t,F_{e},F_{i}\right),$$

where $\epsilon_{\theta }^{\prime }\left(z_{t},t,F_{e},F_{i}\right)$ represents the process guided by the key information $F_{e}$ and $F_{i}$, as well as the model parameter θ. This noise term is used to add details in the inverse process to recover the original face image with high quality. $F_{e}$ and $F_{i}$ represent the conditional guidance used at time step t, which is the key feature information extracted from the pre-trained diffusion model and used to guide the image restoration process. Let θ denote the parameters of the U-Net model, which are optimized during training to improve the quality of the recovered face images.

**Figure 3:**
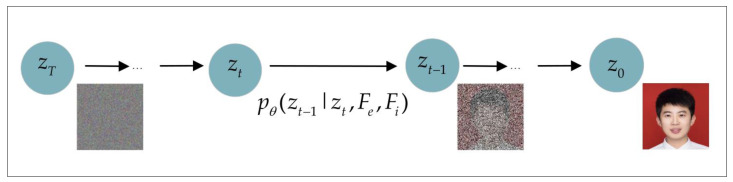
Privacy-protection image denoising recovery process.

Equation (17) is an iterative step used for image generation in the face image restoration process, guides the denoising process of the t−1 step to obtain *x*. By employing latent encoding and raw image guidance, an initial encoding called “denoised” is generated to facilitate the reconstruction of an image that closely resembles the original. This ensures the production of a high-quality restored image, as shown in Figure 3.

(17) $$z_{t-1}^{\prime }=z_{t-1}-\lambda_{t}\nabla z_{0}^{\prime }D_{\theta }\left(x,x_{0}^{\prime }\right),$$

where $z_{0}^{\prime }$ represents the estimated “clean” latent coding derived from $z_{t}$, $\lambda_{t}$ denotes the learning rate and $\nabla z_{0}^{\prime }D_{\theta }\left(x,x_{0}^{\prime }\right)$ represents the loss gradient, indicating the gradient for target $D_{\theta }\left(x,x_{0}^{\prime }\right)$. This loss metric evaluates the similarity between the resulting image and the original.

The initial denoised latent code $z_{0}^{\prime }$ is obtained and then decoded to reconstruct the image representation. This process involves mapping the latent code back to the image space through an inverse transformation, resulting in the recovered image $x^{\prime }$. The decoder takes $z_{0}^{\prime }$ as input and utilizes learned weights and bias parameters to generate a representation that increasingly resembles the original image. The recovered image $x^{\prime }$ is generated using Equation (18).

(18) $$x^{\prime }=Dec\left(z_{0}^{\prime }\right),$$

where $x^{\prime }$ represents the recovered face image after denoising, Dec represents the decoder and $z_{0}^{\prime }$ represents the latent coded image in the latent space.

## 4. Experiment

### 4.1. Implementation Details and Datasets

The experiment was conducted using PyCharm 2021 on a Windows 10 platform. We utilized the CelebA-HQ dataset [35], which consists of approximately 30,000 images with a resolution of 1024 × 1024 pixels. For training purposes, we randomly selected a subset of 12,000 images from CelebA-HQ. Additionally, test images were obtained from a diverse network dataset to simulate real-world scenarios.

The experiment’s performance was evaluated using four metrics: root mean square error (RMSE), mean absolute error (MAE), peak signal-to-noise ratio (PSNR) and structural similarity index (SSIM). Lower values of RMSE and MAE indicate superior image quality, while higher values of PSNR and SSIM suggest the same. The training process employed an NVIDIA RTX 3090 GPU with a batch size of 16. To optimize the outcomes, we utilized the Adam optimizer with a learning rate set to $1\times 10^{-4.5}$ and a weight decay of 0.001.

Root mean square error (RMSE) quantifies the average magnitude of the discrepancy between predicted and actual values, thereby serving as a measure of precision in predictive modeling or estimation.

(19) $$RMSE=\sqrt{\frac{1}{n}\sum_{i=1}^{n}{\left(x_{i}-x_{i}^{\prime }\right)}^{2}},$$

where *n* represents the total number of samples, $x_{i}$ denotes the true value at position *i* and $x_{i}^{\prime }$ signifies the predicted value at position *i*. It represents the average magnitude of the difference between the recovered face image and the original face image.

Mean absolute error (MAE) measures the average absolute deviation between predicted and true values. A lower MAE indicates a reduced discrepancy between predicted and true values, demonstrating improved precision in the predictive model or estimation.

(20) $$MAE=\frac{1}{n}\sum_{i=1}^{n}\left|x_{i}-x_{i}^{\prime }\right|,$$

where *n* represents the total number of samples, $x_{i}$ denotes the true value at position *i* and $x_{i}^{\prime }$ signifies the predicted value at position *i*. It represents the mean absolute deviation between the restored face image and the original face image, indicating the quality of the restoration.

Peak signal-to-noise ratio (PSNR) can be used to evaluate the quality of the recovered images. The higher the PSNR value, the better the quality of the restored image, indicating a closer resemblance to the original image. PSNR is calculated based on the mean squared error (MSE) between the original and restored images.

(21) $$PSNR\left(x_{i},x_{i}^{\prime }\right)=10\cdot log_{10}\left(\frac{255^{2}}{MSE(x_{i},x_{i}^{\prime })}\right),$$

where *x* and $x_{i}^{\prime }$ represent the initial image and the recovered image, respectively.

Structural similarity index (SSIM) measures the similarity between two images by assessing their structure, brightness and contrast. It produces values within the range [−1, 1], where values closer to 1 signify greater similarity and higher image quality.

(22) $$SSIM\left(x_{i},x_{i}^{\prime }\right)=\frac{\left(2\mu_{x_{i}}\mu_{x_{i}^{\prime }}+C_{1}\right)\left(2\sigma_{x_{i}}\sigma_{x_{i}^{\prime }}+C_{2}\right)}{\left(\mu_{x_{i}}^{2}+\mu_{x_{i}^{\prime }}^{2}+C_{1}\right)\left(\sigma_{x_{i}}^{2}+\sigma_{x_{i}^{\prime }}^{2}+C_{2}\right)},$$

where *x* and $x_{i}^{\prime }$ denote the original image and the restored image, respectively. $\mu_{x}$ and $\mu_{x_{i}^{\prime }}$ represent the pixel mean, $\sigma_{x}$ and $\sigma_{x}^{\prime }$ represent the pixel variance, and $\sigma_{xx_{i}^{\prime }}$ represents the covariance of the pixel. The constants $C_{1}$ and $C_{2}$ are used to stabilize the denominator, typically taking values such as $C_{1}={\left(K_{1}\times L\right)}^{2}$ and $C_{2}={\left(K_{2}\times L\right)}^{2}$, where $K_{1}=0.01$, $K_{2}=0.03$, *L* = 255. Indicates the structural similarity between the recovered face image and the original face image.

### 4.2. Compare with Other Methods

In this section, we conduct experiments to evaluate the efficacy of our proposed method for facial privacy-protection and -recovery performance compared to existing approaches. Specifically, we compare our method with those proposed by Maximov et al. [36], Shan et al. [37], Hukkelas et al. [38], You et al. [39], Yang et al. [40], Li et al. [41], Gu et al. [42] and He et al. [43]. Hukkelas et al. [38] utilize a GAN-based approach to generate realistic and anonymous images for individual privacy protection. Maximov et al. [36] employ a GAN-based model to anonymize identity information through conditional generation. Shan et al.’s method focuses on adversarial examples to preserve face privacy while maintaining visual fidelity. You et al. [39] propose reversible face recognition using a reversible mosaic transformation for privacy protection. Yang et al. introduce a reversible mask network for achieving reversible face privacy protection. The Li et al. [41] method aims at reversible identity concealment with diverse concealment processes through latent encryptors. The Gu et al. [42] method combines password-based identity hiding and restoration. He et al. [43] achieve face identity hiding through conditional embedding policies. The following sections present the results of the comparison in terms of both qualitative and quantitative experiments.

Qualitative privacy preservation in qualitative experiments refers to the effectiveness of face privacy preservation methods as perceived by human observers. To evaluate the effect of privacy-protection measures from a qualitative perspective, we emphasize the naturalness of the processed images and the inability to identify the original individuals. Quantitative experiments refer to evaluating the performance of face privacy protection by quantifying the difference between the privacy protection-generated image and the original image through numerical data.

#### 4.2.1. A Qualitative Comparison of the Privacy-Protection Effect of Faces

**Figure 4:**
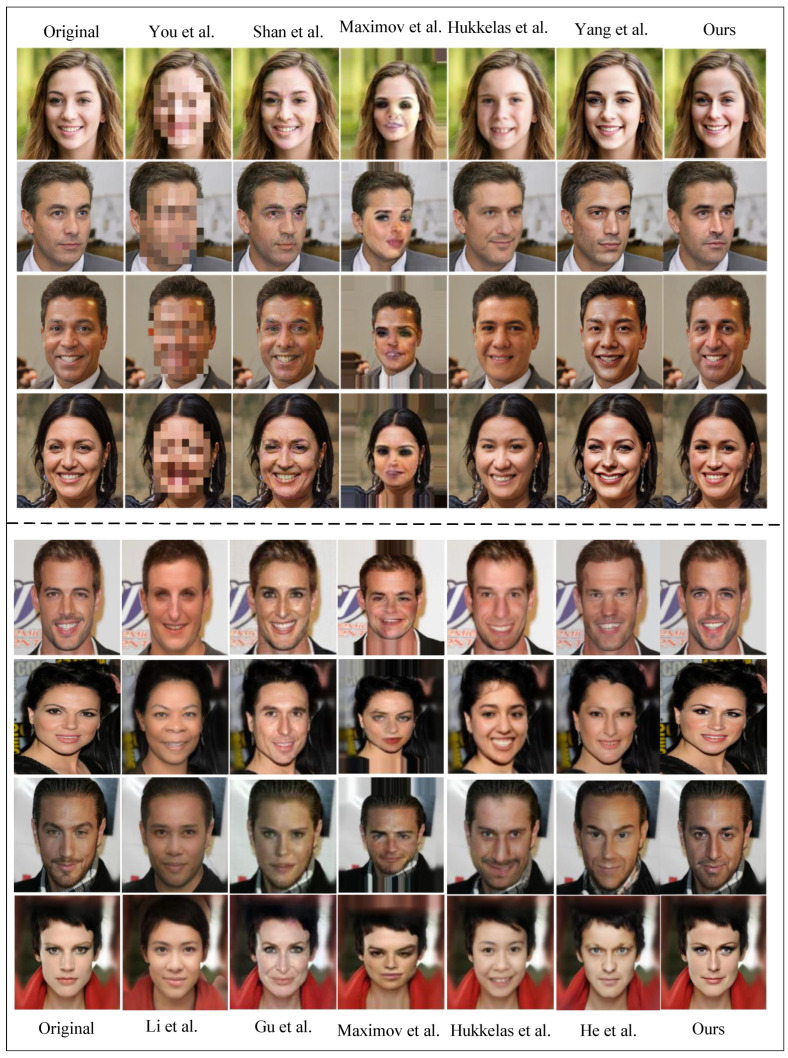
A qualitative comparison of the effect of face privacy protection with other methods. Maximov et al. [36], Shan et al. [37], Hukkelas et al. [38], You et al. [39], Yang et al. [40], Li et al. [41], Gu et al. [42] and He et al. [43].

For face privacy protection, whether the processed image is natural and not discovered by attackers is an important evaluation index. In this section, we evaluate the visual quality and facial privacy-protection effectiveness of various methods through subjective visual comparison. Eight face images of different styles from the web were randomly selected for evaluation. In Figure 4, the first column shows the original face image that needs to be protected, and the subsequent columns represent the privacy-preserving images generated by other methods, respectively. The final column showcases the images generated by our proposed method. Visual inspection reveals noticeable visual distortions in the images processed by You et al., Maximov et al. [36], Shan et al. [37], Hukkelas et al. [38] and He et al. [43]. Gu et al.’s method [42] generates faces with distinctly different identities but suffers from unsatisfactory visual quality and mismatched attributes. Li et al.’s method produces a wide variety of faces but may introduce unnatural features in some cases. Although Yang et al.’s method maintains good visual fidelity, it may introduce information loss due to the retention of reversibility, leading to diminished realism. Our proposed method outperforms others in terms of visual quality, facial privacy-protection effectiveness and naturalness, preserving the posture and expression of the original image while achieving better photorealism.

#### 4.2.2. Quantitative Comparison of Face Privacy-Protection Effect

The effectiveness of face privacy protection is assessed in this section by comparing the success rates of protection against face recognition. We randomly selected 2000 face images from the CelebA-HQ dataset [35] as protected face images and another 2000 face images with diverse identities and styles as bootstrap conditions. Subsequently, our proposed method was used to generate 2000 face privacy-preserving images. All processed face images underwent testing with two types of face-recognition tools: commercially available tools and academically researched tools. Specifically, evaluation was conducted using Baidu Intelligent Cloud’s [44] framework for facial recognition along with the renowned and reliable face-recognition API, FaceNet [45], which has achieved top rankings in authoritative public evaluations.

The equation for calculating the protection success rate can be defined as follows,

(23) $$PR=\frac{NP}{NT}\times 100\%,$$

where NP represents the number of faces that cannot be recognized after protection. NT denotes the total number of faces. If a privacy-protection method can make the face-recognition system unable to identify the identity of the person in the image, the success rate of this method will be high.

The results are summarized in Table 1. Notably, Shan et al.’s method [37] exhibits protection success rates of 76.5% and 23.6%. This lower success rate can be attributed to its reliance on adversarial interference, which may not be compatible with the loss functions and training methodologies employed by many commercial face-recognition tools. Conversely, methods proposed by You et al. [39], Maximov et al. [36], Hukkelas et al. [38], Yang et al. [40] and our proposed method achieved protection success rates of 100% when tested using the face-recognition tool from Baidu Intelligent Cloud [45]. Notably, You et al.’s method employs reversible mosaic transformation, rendering the processed faces unrecognizable by Baidu Intelligent Cloud [45]. Maximov et al. [36] and Hukkelas et al. [38] modify and replace original facial features, leading to inaccuracies in face identification by Baidu Intelligent Cloud [45] although the original identity remains unrecoverable. Yang et al.’s method utilizes a reversible mask network for privacy protection but lacks diversity in generated images. In contrast, our proposed method demonstrates superior protection-recognition rates compared to other privacy-protection methods in the FaceNet [44] face-recognition framework, even outperforming You’s approach after removing all mosaic transformations. In summary, our approach offers enhanced security measures.

#### 4.2.3. Qualitative Comparison of the Restorative Effect of the Face

**Figure 5:**
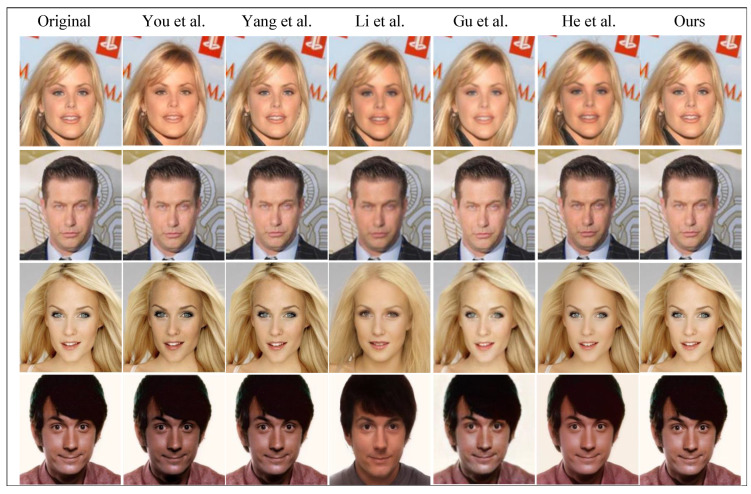
A qualitative comparison of the effect of face privacy protection with other methods. You et al. [39], Yang et al. [40], Li et al. [41], Gu et al. [42] and He et al. [43].

As mentioned earlier, achieving high-fidelity restoration of the original face is the primary objective of this study to suit new face privacy-protection scenarios. Recoverability serves as a vital criterion for evaluating face privacy-protection methods. Table 2 provides a comparative analysis of the reversibility of these methods. Notably, among the eight comparative methods discussed earlier, You et al.’s method, Yang et al.’s method, Li et al.’s method [41], Gu et al.’s method [42] and He et al.’s method [43] are reversible. Figure 5 presents a qualitative comparison, indicating that the recovered images produced by our method exhibit a smoother, sharper and closer resemblance to the original images. In summary, our method effectively safeguards the privacy of protected faces while offering near-perfect recovery of obscured faces.

#### 4.2.4. Quantitative Comparison of Face Reversibility Effects

Utilizing the recovered images depicted in Figure 5, we computed the similarity metrics between the original and recovered images. Specifically, we calculated the PSNR, SSIM, RMSE and MAE values between the protected and recovered surfaces. Table 3 presents a quantitative comparison of these metrics.

The results in Table 3 demonstrate that our method outperforms existing reversible methods in identity recovery across various evaluation metrics.

We used the recovered face images for the face-recognition model ArcFace as well as Facenet to verify the recovery effect on the face-recognition model. We evaluate the effectiveness of the face recovery effect by comparing the recognition rates, as illustrated in Table 4.

As can be seen from the table, in the Facenet face-recognition model, except for You et al., the other methods have a 100% recognition rate. However, in the ArcFace face-recognition model, only He et al.’s method and our method achieve a 100% recognition rate. Combining with the various image recovery indicators in Table 3, our method is better than He et al.’s method in all indicators. At the same time, it can efficiently restore the original identity, which has more advantages in terms of privacy protection.

### 4.3. Ablation Study with Other Methods

#### 4.3.1. Add the Influence of Identity and Style-Guided Conditionality

**Figure 6:**
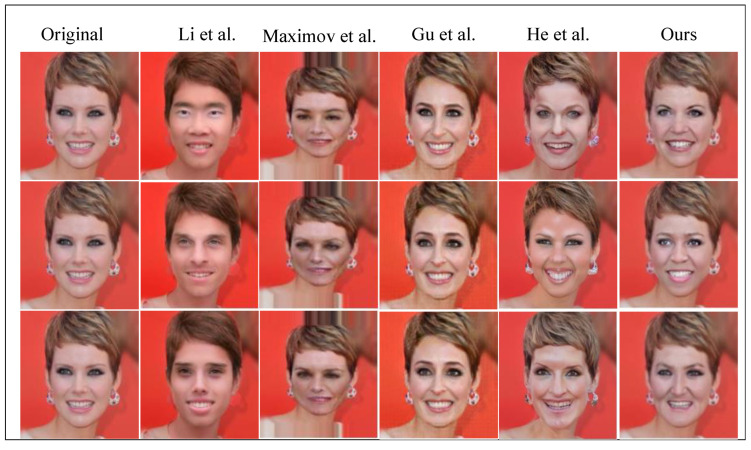
Visual comparison of generated results guided by multiple conditions in privacy protection. Maximov et al. [36], Li et al. [41], Gu et al. [42] and He et al. [43].

In our approach, we employ multiple guidance conditions, including identity and style guidance, to provide comprehensive control for a single diffusion model. This contrasts with methods that rely on single-guidance information. To validate the advantages of employing multiple conditional controls over a single set of bootstrap information, we conducted experiments. Figure 6 illustrates the outcomes of employing multiple conditional controls using identity and style guidance compared to utilizing only single bootstrap information. The results demonstrate that while various methods, such as Gu et al. [42], Maximov et al. [36], Li et al. [41] and He et al. [43], can generate faces, they exhibit limitations. For instance, faces generated by Gu et al. [42] exhibit common characteristics across different passwords; Maximov et al. [46] produce faces with low quality and noticeable stitching marks; Li et al. [41] yield facial features that are similar when encrypting different faces with the same password; and the faces generated by He et al. [43] lack realism. In contrast, our method, leveraging multiple conditional controls guided by identity and style, demonstrates robust decoupling and editability, resulting in the generation of diverse and realistic face images capable of achieving varying degrees of privacy protection.

**Figure 7:**
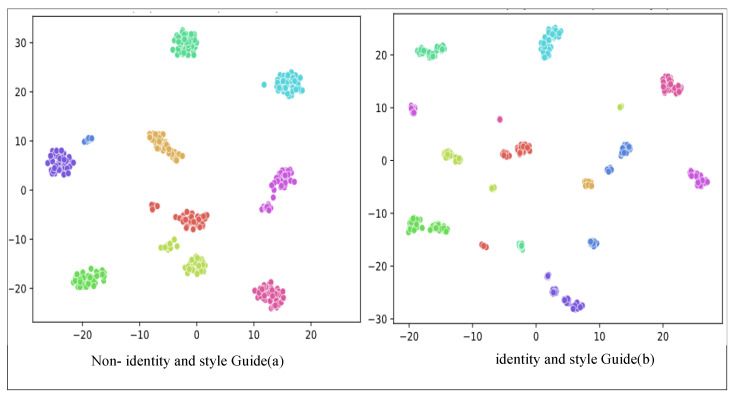
T-SNE visualization results.

In Figure 7, we utilized a face-recognition network to extract conditional control information from the generated face images. We employed t-SNE dimensionality reduction [46] and conducted a visualization experiment to compare the outcomes of multiple conditional controls with identity and style guidance against those without conditional control. Figure 7a illustrates that identity clusters are relatively close, with distinct clusters well separated in the hyperplane. Conversely, Figure 7b demonstrates that the privacy-preserving faces we generated exhibit greater dispersion, occupying a larger portion of the hyperplane and offering a wider diversity of face images.

#### 4.3.2. The Effect of Realistic Control

**Figure 8:**
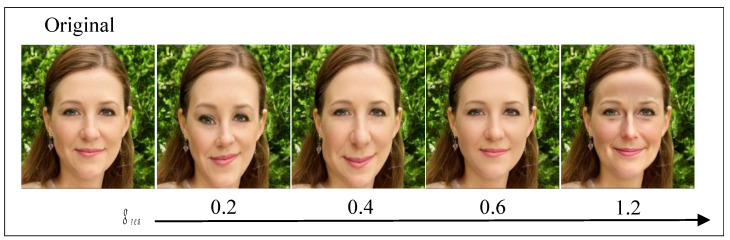
The impact of the scale factor in photorealism on the generated outcomes.

In the privacy-protection experiment, we designed a photorealistic control strategy experiment to assess its effectiveness and its impact on the generated results across different degrees. Specifically, we varied the realism scale factor $g_{rea}$ in the realism control and experimentally evaluated its influence. The outcomes are depicted in Figure 8. As illustrated, decreasing $g_{rea}$ results in more retained high-frequency detail in the generated face image, with greater variations leading to increased distortion. Conversely, increasing $g_{rea}$ yields smoother face images that closely resemble real faces. Optimal similarity and realism with real faces are observed when $g_{rea}\in \left(0.5–0.7\right)$. Therefore, in our methodology, to ensure the best privacy protection and user visual experience, we select a realism scale factor of $g_{rea}=0.6$ for our photorealistic control strategy. This choice not only obscures sensitive information but also effectively guides control with dual conditions of identity and style, resulting in privacy-preserving faces that closely resemble real faces, thereby enhancing authenticity and achieving privacy protection.

#### 4.3.3. The Effect of Noise Intensity

To assess the robustness of the restored image, we introduce controlled random noise into the face image restoration process and vary the scale factor $S_{n}$ to examine its impact on restoration outcomes. Table 5 presents the results obtained with different noise scale factors. When decreasing noise intensity (i.e., reducing the scale factor $S_{n}$), the outcomes gradually resemble the original image more closely. Specifically, when $S_{n}=0.8$, the performance is optimal. By ensuring that identity and style attributes remain unchanged, our model’s performance can be effectively enhanced to achieve privacy protection. Consequently, we select $S_{n}=0.8$ as the noise scale factor, which effectively obscures sensitive information while introducing significant changes to optimize our model’s performance.

## 5. Conclusions

To address evolving societal trends towards greater self-expression while safeguarding facial privacy and preserving high-fidelity facial features, we propose a novel reversible face privacy-protection method based on a diffusion model(DIFP). Our method comprises three key parts. Part 1 involves generating facial images using a classifier-free guided diffusion model with multifaceted control over identity and style. Part 2 focuses on the iterative refinement of variables to enable control over identity, style and realism during image generation to achieve privacy protection. Part 3 employs denoising techniques to perform identity recovery, resulting in visually distinct facial features that can be almost entirely recovered by authorized users. Extensive experimentation demonstrates the effectiveness of our method both quantitatively and qualitatively. Future research directions may focus on comprehensive privacy protection in multi-modal scenarios such as images, videos and audio to ensure effective safeguarding of individuals’ identities and private information across various data types.

## Figures and Tables

**Table 1:** Quantitative comparison of the effect of face privacy protection with other methods.

Method	Business Framework	FaceNet
You et al. [39]	100%	100%
Shan et al. [37]	76.5%	23.6%
Maximov et al. [36]	100%	84.9%
Hukkelas et al. [38]	100%	92.3%
Yang et al. [40]	100%	96.3%
Li et al. [41]	-	98.5%
Gu et al. [42]	-	95.7%
He et al. [43]	-	98.8%
Ours	100%	99.2%

**Table 2:** Whether there is a recoverability comparison.

Method	Reversibility
You et al. [39]	√
Shan et al. [37]	×
Maximov et al. [36]	×
Hukkelas et al. [38]	×
Yang et al. [40]	√
Li et al. [41]	√
Gu et al. [42]	√
He et al. [43]	√
Ours	√

**Table 3:** Comparison results between other methods and our method with regard to the reversibility effect.

Method	RMSE ↓	MAE ↓	SSIM ↑	PSNR ↑
You et al. [39]	14.133	2.972	0.494	13.527
Yang et al. [40]	0.527	0.496	0.523	18.649
Li et al. [41]	0.212	0.148	0.597	19.489
Gu et al. [42]	0.177	0.115	0.762	28.693
He et al. [43]	0.085	0.054	0.854	28.900
Ours	0.046	0.027	0.938	30.174

**Table 4:** Compare the recognition rates of face restoration effects on face-recognition models.

Method	FaceNet	ArcFace
You et al. [39]	98.6%	91.7%
Yang et al. [40]	100%	93.3%
Li et al. [41]	100%	96.2%
Gu et al. [42]	100%	98.5%
He et al. [43]	100%	100%
Ours	100%	100%

**Table 5:** Results of comparison with other methods in terms of noise intensity.

Noise Scale Factor	RMSE ↓	MAE ↓	SSIM ↑	PSNR ↑
2	0.074	0.049	0.667	28.627
1.2	0.053	0.036	0.894	29.335
0.8	0.046	0.027	0.938	30.174
0.5	0.049	0.033	0.915	27.156

## Data Availability

All training datasets used in this article are from public datasets on the Internet.
